# Do diagnostic delays in cancer matter?

**DOI:** 10.1038/sj.bjc.6605384

**Published:** 2009-12-03

**Authors:** R D Neal

**Affiliations:** 1 Department of Primary Care and Public Health, North Wales Clinical School, Cardiff University, Gwenfro 5, Wrexham Technology Park, Wrexham LL13 7YP, UK

**Keywords:** diagnosis, symptoms, primary care, delays

## Abstract

**Background:**

The United Kingdom has poorer cancer outcomes than many other countries due partly to delays in diagnosing symptomatic cancer, leading to more advanced stage at diagnosis. Delays can occur at the level of patients, primary care, systems and secondary care. There is considerable potential for interventions to minimise delays and lead to earlier-stage diagnosis.

**Methods:**

Scoping review of the published studies, with a focus on methodological issues.

**Results:**

Trial data in this area are lacking and observational studies often show no association or negative ones. This review offers methodological explanations for these counter-intuitive findings.

**Conclusion:**

While diagnostic delays do matter, their importance is uncertain and must be determined through more sophisticated methods.

It is now well established that the United Kingdom has poorer cancer outcomes compared with much of Western Europe (Berrino *et al*, 2007). The consensus is that one of the major reasons for this is more advanced stage at diagnosis. One solution to this problem is to reduce diagnostic delays on the premise that this will lead to earlier stage diagnosis and improved outcomes. This paper will contextualise the diagnostic process and critically appraise the evidence that examines the association between delays and cancer outcomes.

Tumours typically grow progressively, with the ‘doubling time’ (a recognised period of time that it takes for the tumour to double in size) being a key measure of speed of tumour growth. This varies between types of tumour but even within tumour types, there can be significant variation, leading to unpredictable differences in patterns of symptoms and symptom complexes, speed of onset and progression of symptoms (Ford and Mitchell, 1999).

Around 90% of cancers will present symptomatically (Hamilton, 2008). There is a prevailing hypothesis within primary care that most patients have symptoms that are either self-limiting or represent chronic and benign disease until proven otherwise. In cancer this may be counter-productive, and creates a dilemma for primary care. On one hand, health professionals, whether they are general practitioners (GPs) or practice nurses (who do much chronic disease management and monitoring), have to be vigilant for ‘alarm symptoms’ (Jones *et al*, 2007) that are rarely caused by cancer. On the other hand, they are aware that most potential cancer symptoms are almost exactly the same as those of common chronic or minor diseases. Furthermore, there may be difficulty in assessing the positive predictive value of symptoms and what they really mean, and placing these within the current National Institute for Health and Clinical Excellence (NICE) urgent suspected referral guidelines (NICE, 2005). These have been shown to have a low predictive value in determining cancer (Allgar *et al*, 2006) and may prioritise patients who stand to gain least from urgent referral in terms of survival (Neal *et al*, 2007).

Hence, the vexed question of what can be done to reduce delays is of paramount importance. The need to determine the exact relationship between symptom duration and clinical outcomes (usually survival, but sometimes stage as a proxy for survival) is essential. Delays may occur at any stage of the diagnostic cancer journey. There may be ‘patient delays’ where the patient may not recognise suspicious cancer symptoms or act on them. There may also be ‘primary care delays’, where there are unnecessary delays in the recognition, onward referral of or investigation for suspicious symptoms. After this, there may be ‘system delays’ where there may be a considerable wait for either non-urgent referrals (as is the case for the majority of cancer patients) or GP-initiated diagnostic investigations. Finally, ‘secondary care delays’ are where there may be prolonged time spent in secondary care before diagnosis, sometimes due to patients being investigated in the ‘wrong’ specialty.

While a small element of delay is inherently inevitable in all cancer diagnostic pathways, it is likely that in a significant percentage of patients there is considerable preventable delay. The important question is how much of this preventable delay leads to poorer outcomes. If people could be diagnosed earlier, what difference would this make to survival and other clinical outcomes?

## Scoping review of the literature

The existing literature in this area is very mixed and, at times, confusing. There is a dearth of trial evidence (e.g. of interventions to reduce delays). Indeed, recent systematic reviews examining factors associated with delay in colorectal and upper gastrointestinal cancers identified no trials at all (Macdonald *et al*, 2006; Mitchell *et al*, 2008). Most of the published literature reports data, inevitably, from observational studies. It may seem logical that outcomes should worsen with longer symptom duration, but there are a number of observational studies that seem to show the opposite, across a wide range of cancers. How and why should this be so? A major systematic review of the world literature on this topic is due to report in early 2010. As a prelude to this, a scoping review identified evidence from 47 studies in 13 different cancers; some studies reported findings for more than one cancer in a single paper (Table 1). These included two systematic reviews: one well-conducted review for breast cancer that showed a clear association between shorter symptom durations and better outcomes (Richards *et al*, 1999); and one less rigorous review for lung cancer that showed a weakly positive association (Jensen *et al*, 2001). Of the 45 other studies, 9 showed a positive association, 9 showed a negative association (i.e. shorter symptom durations were associated with worse outcomes) and 29 showed no association.

A close examination of this body of literature has identified multiple methodological issues that need to be taken into consideration when trying to determine the association between symptom duration and clinical outcomes. These will be discussed in turn.

### Different definitions of delays

Different studies have used different definitions of delay, making comparison between studies and settings very difficult. Some of this is inevitable, given the different structures of international health services, including the varying roles of primary care, and the access of patients directly to secondary care specialists and diagnostic investigations. However, there seems to be some broad consensus that ‘patient delay’ may occur in the time period from first experience of a potential cancer symptom to telling a health professional about it. Similarly, there is broad consensus that ‘primary care delay’ can occur in the time period from first presentation of the symptom to onward specialist referral to GP-initiated diagnostic investigation. ‘System delay’ would occur in the time that it then takes for these investigations to happen, and delays in ‘secondary care’ happen during the time from first being seen in secondary care to diagnosis. Some papers also report ‘treatment delay’ – the time from diagnosis to first treatment.

### Different ways of measuring delays

There are many ways of measuring delays and each has its own problems. Questions can be asked of the patient, the health professional or the medical record(s). Patient-centred studies are most likely to represent truer ‘patient delays’, but the answers that patients give vary enormously depending on how the questions are asked. In recent years, in-depth qualitative studies have shown that patients report symptoms attributable to their cancer for much longer than previously thought, as in, for example, lung (Corner *et al*, 2005) and ovarian cancers (Bankhead *et al*, 2005). Surveys with closed-response questions generally report much shorter delays, as is the case for data from the NHS cancer survey (Allgar and Neal, 2005). Additionally, any interview or patient-completed survey is prone to recall bias, which is hard to avoid.

Studies that use medical records to examine diagnostic delays have advantages in that records are made contemporaneously, and are not prone to recall bias. However, they are prone to missing data, which is often non-random. For example, primary care records are more likely to contain an entry for abdominal pain if the clinician thinks that the pain is significant. If they do not, the pain may not have been recorded even though it was presented. Studies using records are good at capturing events such as referrals and investigations, but doing so precisely for diagnosis can be difficult. When exactly is a diagnosis made: when a pathological specimen is reported, when the multi-disciplinary team meets, when patients are told or when a diagnosis confirmation letter is coded in primary care? This can cause difficulty and errors but there are algorithms for standardising this (Tate *et al*, 2009).

The significance of these difficulties is that the context for how delay is counted and captured needs to be understood. Until then, comparisons between studies may be impossible or give very conflicting results.

### Difficulties in comparing cancers that behave very differently

It is difficult to make between-cancer comparisons given the variation in how they present and grow. What may hold for breast cancer may have no bearing on colorectal, lung or prostate cancer. Interventions aimed at reducing the duration of the diagnostic pathway are likely to be very different between different cancers and what works for one may well not work for another. However, there are some findings that may have relevance for different cancers.

### Difficulties in measuring outcomes

Survival is the most important outcome. While it is the primary outcome in some parts of the literature, others use different measures as a proxy for survival. These include stage (which correlates well with survival), and different treatment options (such as eligibility for potentially curative treatments). Health-related quality of life measures are also of importance. The published literature reports a variety of outcome measures, and this can make comparison between studies difficult.

### Failure to account for speed of growth of tumours

Tumours of a single cancer type can appear to be similar but grow at very different rates and with different levels of aggressiveness. A fast-growing tumour is likely to cause symptoms with more rapid progression, leading to a quicker diagnostic journey but worse outcomes because of aggressive growth and spread. Conversely, a slower-growing tumour is likely to cause symptoms that develop more insidiously and take longer to diagnose. However, the outcome may be better, given that curative life-prolonging treatments may be offered. Hence, there is what is sometimes called a ‘paradox’ (although it is really nothing of the sort) where patients with shorter delays may do worse than those with longer ones. This phenomenon has been described in several cancers, for example, endometrial (Crawford *et al*, 2002) and colorectal (Rupassara *et al*, 2006), but is likely to exist in more. It also probably explains why the negative associations reported in some colorectal cancer studies disappear when the data are corrected for emergency admissions (Stapley *et al*, 2006).

The issue for the body of literature looking at the association between delay and outcome is that many studies have not allowed for differences in the speed of tumour growth in their analysis. Many have simply analysed data for all patients together. It seems hardly surprising therefore that many studies have shown equivocal or negative findings.

### Confounding effect of lead-time bias

Lead-time bias is the bias that may occur when outcomes are compared, but where the onset of measuring ‘delay’ is different as a result of diagnosis earlier in the natural history of the cancer, but that will have no effect on the outcome. The Richards *et al* (1999) systematic review of breast cancer reported that only 4 out of 87 included studies took lead-time bias into account.

## Conclusion

In conclusion, diagnostic delays in cancer do matter, but it is hard to quantify their impact on survival or mortality. The ‘amount’ that they matter is clear in breast cancer but far less so in other cancer types. More empirical work is needed to determine the importance of delays and, in particular, the likely effect of interventions to reduce delay in specific parts of the diagnostic journey. More work is also needed to determine the effect of delays between diagnosis and first treatment.

There are significant windows of opportunity to reduce symptom duration through:

- Reducing patient delay by increasing awareness of symptoms and the understanding of how and when to act on these.
- Reducing primary care delay by increasing awareness of potential cancer symptoms among primary care clinicians, by changing culture towards one where potential cancer symptoms are considered suspicious until proven otherwise, and by lowering the thresholds for referral or requesting GP-initiated investigations.
- Reducing system delay, by revising and implementing new urgent cancer referral guidance. These must account for the considerable body of primary care-based research on the meaning of symptoms and symptom complexes that has been published since the last update (NICE, 2005). More importantly, faster-track pathways are needed for diagnostic investigations and for patients with potential cancer symptoms that do not fulfil the urgent referral criteria. There is also a need for innovations to reduce unnecessary delay, such as proceeding straight to computerised tomography scan for a suspicious chest X-ray, rather than referring back to the GP or directly to the chest clinic.

Working to reduce delays in cancer diagnosis in these ways will contribute to the ultimate goal of achieving earlier stage diagnosis with its associated options for curative or life-prolonging treatment.

## Figures and Tables

**Table 1:** Summary of findings of scoping review

Cancer site	Evidence (‘positive’=longer delay associated with poorer outcomes, ‘negative’=longer delay associated with better outcomes)
Breast	Systematic review: 1 positive study within this scoping review (Richards *et al*, 1999) Subsequent studies: 1 positive (Arndt *et al*, 2002), 1 negative (Sainsbury *et al*, 1999)
Lung	Systematic review: 1 weakly positive study within this scoping review (Jensen *et al*, 2001) 5 subsequent studies: 3 negative (Yoshimoto *et al*, 2002; Myrdal *et al*, 2004; Neal *et al*, 2007) 2 no association (Koyi *et al*, 2002; Quarterman *et al*, 2003)
Colorectal	10 no association (Robinson *et al*, 1984; Stubbs and Long, 1986; Kyle *et al*, 1991; Majumdar *et al*, 1999; Roncoroni *et al*, 1999; Young *et al*, 2000; Kiran and Glass, 2002; Gonzalez-Hermoso *et al*, 2004; Bharucha *et al*, 2005; Neal *et al*, 2007) 4 negative (Mulcahy and O'Donoghue, 1997; Langenbach *et al*, 2003; Olsson *et al*, 2004; Rupassara *et al*, 2006), however, for three of these, the association disappears if data are corrected for emergency admissions (Mulcahy and O'Donoghue, 1997; Olsson *et al*, 2004; Rupassara *et al*, 2006)
Melanoma	5 no association (Krige *et al*, 1991; Blum *et al*, 1999; Oliviera *et al*, 1999; Brochez *et al*, 2001; Baade *et al*, 2006) 1 weakly positive (Betti *et al*, 2003)
Upper gastrointestinal	1 no association (oesophageal; Kotz *et al*, 2006) and 2 no association (gastric; Martin *et al*, 1997; Porta *et al*, 1991) 1 positive (oesophageal; Martin *et al*, 1997) and 1 weakly positive (oesophageal; Porta *et al*, 1991)
Ovarian	3 no association (Robinson *et al*, 1984; Kirwan *et al*, 2002; Neal *et al*, 2007) 1 positive (Wikborn *et al*, 1996)
Endometrial	1 negative (Crawford *et al*, 2002) 1 no association (Menczer *et al*, 1995)
Testicular	1 positive (Hernes *et al*, 1996) 1 no association (Toklu *et al*, 1999)
Bladder	2 no association (Robinson *et al*, 1984; Liedberg *et al*, 2003)
Prostate	1 no association (Neal *et al*, 2007)
Oropharyngeal	1 positive (Pitchers and Martin, 2006)
Laryngeal	1 positive (Teppo *et al*, 2003)
Retinoblastoma	1 no association (Goddard *et al*, 1999)
